# Measuring the expansion of myocardial extracellular volume using quantitative perfusion imaging: Validation with T1 mapping and simultaneous ^18^FDG-PET in patients after acute myocardial infarction

**DOI:** 10.1186/1532-429X-17-S1-P232

**Published:** 2015-02-03

**Authors:** Karl P Kunze, Christoph Rischpler, Sylvia Schachoff, Markus Schwaiger, Stephan G Nekolla

**Affiliations:** Department of Nuclear Medicine, Klinikum Rechts der Isar, Munich, Germany

## Background

Expansion of myocardial extracellular volume (ECV) as detected by native and post contrast T1 mapping has been associated with different types of cardiomyopathy. We hypothesize that fully quantitative evaluation of MR perfusion imaging can localize and quantify edematous ECV expansion after acute coronary events with sensitivity equivalent to ECV measurements based on T1 mapping or the detection of corresponding inflammatory and metabolic processes with ^18^FDG-PET.

## Methods

Imaging was performed on a 3T PET/MR scanner in 10 patients following coronary obstruction and subsequent revascularization (intervention/imaging: 5.2±0.4 days). For T1 mapping, a Modified Look-Locker Inversion recovery sequence (MOLLI) was applied in free breathing with retrospective motion correction. Post contrast T1 maps covering three left ventricular short axis slices were acquired 25±4 minutes after a 0.2 mmol/kg infusion of Gd-DTPA. ECV maps were created after registration of native and post contrast T1 maps using individual hematocrit values (38.9±2.7 %). Perfusion imaging was performed with a 0.1 mmol/kg Gd-DTPA bolus using an ECG-triggered saturation recovery FLASH with dual sequence design and retrospective motion correction. Perfusion data were acquired for 90 cardiac cycles covering the same slices with the same resolution as the MOLLI acquisition. A Fourier space implementation of an axially distributed (DP) model was used to calculate plasma flow and extracellular distribution volume. ^18^FDG images were acquired in fasted condition to suppress unspecific uptake. Perfusion results were compared quantitatively and sector wise to ECV derived from T1 mapping and qualitatively to ^18^FDG images in one mid ventricular slice per patient using standard AHA segmentation.

## Results

**Figure 1:**
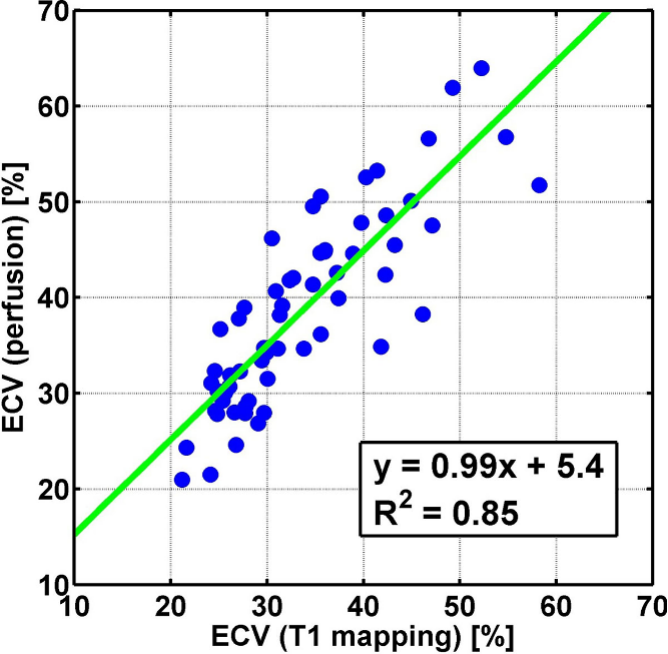
Correlation plot comparing ECV measurements based on T1 mapping with ECV measurements based on perfusion imaging

Sector wise ECV measurements based on T1 mapping and perfusion modeling correlated (R2=0.85) while perfusion imaging overestimated ECV by a constant 5.4% compared to T1 mapping (fig. 1). Myocardial plasma flow showed no correlation with ECV from T1 mapping (R2=-0.15). Agreement between ^18^FDG uptake and ECV expansion derived from perfusion imaging and T1 mapping was excellent in all patients.

**Figure 2:**
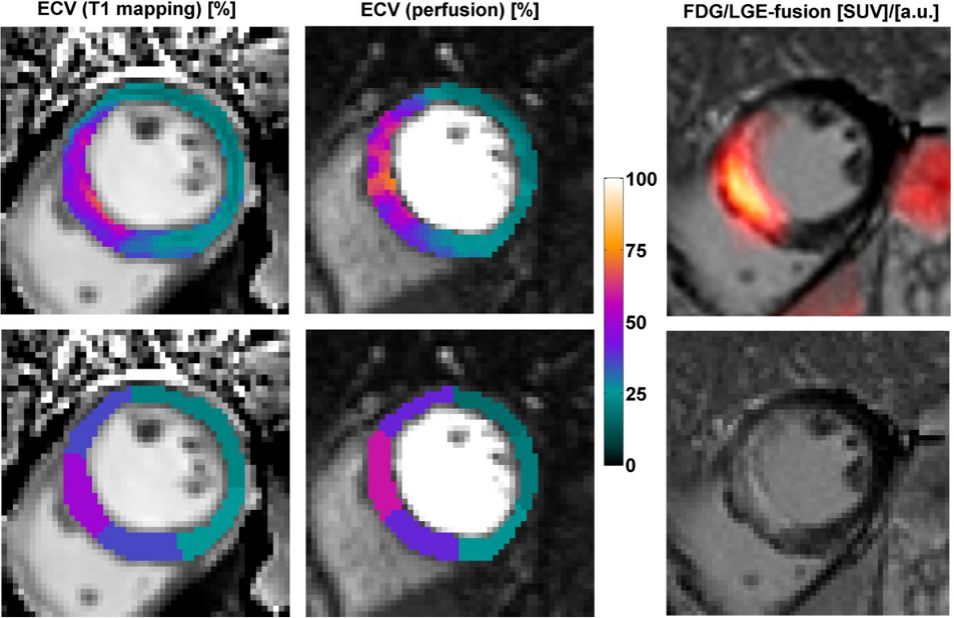
Example slice showing pixel wise and sector wise evaluation of ECV from T1 mapping and perfusion imaging in the left and center columns. The upper right image shows ^18^FDG uptake, the lower right image shows consistency with Late Gadolinium Enhancement

## Conclusions

Detection of ECV expansion using perfusion imaging together with advanced modeling approaches exceeding the scope of first pass perfusion is equivalent in sensitivity to ECV measurements based on T1 mapping. A small constant overestimation of perfusion-derived ECV compared to T1 mapping is attributed to partial volume effects degrading the arterial input function in the dual sequence approach. These findings validate for the first time the ability of myocardial perfusion imaging to detect ECV expansion, while offering the possibility to quantify ischemia and other hemodynamic parameters simultaneously. The interpretation of ECV expansion as an indicator of inflammatory processes is strongly supported by the results of a simultaneously performed ^18^FDG-PET examination.

